# Increased dietary acid load May elevate the risk of coronary artery disease severity: Findings from a cross-sectional study

**DOI:** 10.1016/j.ijcrp.2025.200423

**Published:** 2025-05-15

**Authors:** Zeinab Ghorbani, Fatemeh Dashti, Zahra Saeedirad, Amir Aris, Marjan Mahdavi-Roshan, Arsalan Salari

**Affiliations:** a Cardiovascular Diseases Research Center, Department of Cardiology, Heshmat Hospital, School of Medicine, Guilan University of Medical Sciences, Rasht, Iran; b Department of Clinical Nutrition, School of Medicine, Guilan University of Medical Sciences, Rasht, Iran; c Department of Clinical Nutrition, School of Nutritional Sciences and Dietetics, Tehran University of Medical Sciences, Tehran, Iran; d Department of Clinical Nutrition and Dietetics, Faculty of Nutrition and Food Technology, Shahid Beheshti University of Medical Sciences, Tehran, Iran

**Keywords:** Atherosclerosis, Coronary heart disease, Diet acidity

## Abstract

**Background:**

Chronic low-grade metabolic acidosis appears to play a role in the development of chronic disorders. This study aims to examine the relationship between Potential Renal Acid Load (PRAL) and Net Endogenous Acid Production (NEAP) and the risk of severe coronary artery disease (CAD) in participants undergoing elective angiography.

**Methods:**

In this cross-sectional study, the data of 895 participants from the Nutrition Heshmat Registry (NUTHER) was collected. Dietary data were obtained using a validated food frequency questionnaire to calculate PRAL and NEAP. Participants were categorized into severe CAD (Gensini score≥60; n = 526) and non-severe CAD (Gensini score<60; n = 369). Logistic regression was conducted to evaluate the odds ratio (OR) and 95 % confidence interval (95 %CI). Restricted cubic spline (RCS) regression was employed to explore potential nonlinear associations between PRAL, and NEAP and severe-CAD risk.

**Results:**

After adjusting for potential confounding factors, participants in the third to fourth quartiles of energy-adjusted PRAL and NEAP exhibited higher odds of severe CAD that were approximately 1.62–1.80 times and 1.67–2.76 times greater, respectively, compared to those in the 1^st^quartiles (4^th^quartile ORs(95 %CI) for: PRAL: 1.62 (1.05, 2.51); and NEAP: 1.67 (1.07, 2.61) (P-for-trend<0.021). RCS analysis showed a linear dose-response relationship between elevated PRAL and severe CAD risk (P-for-overall-trend = 0.0176; P-for-nonlinearity = 0.1552), and a nonlinear association between higher NEAP and increased severe CAD risk (P-for-overall-trend = 0.0001; P-for-nonlinearity = 0.006).

**Conclusion:**

The findings indicate a significant association between higher dietary acid load and increased risk of severe CAD, suggesting that a more acidic diet may contribute to the progression of atherosclerosis. However, further prospective studies are necessary to validate these observations.

## 1 Introduction

Cardiovascular disease (CVD) encompasses of broad disorders namely hypertension, ischemic heart disease, heart failure, and coronary artery disease (CAD) [1,2]. As the primary cause of mortality and morbidity globally, CAD also accounts for approximately 50 % of yearly deaths in Iran [3,4]. CAD is characterized as an inflammatory disorder stemming from intricate and multifactorial processes. These processes include the development of atherosclerosis and the subsequent formation of atherosclerotic thrombosis within the coronary arteries. This complexity highlights the interplay of various risk factors and pathophysiological mechanisms that contribute to the progression of CAD, such as gender, age, family history, and genetics, or modifiable ones like obesity, lipid profile, smoking, and diet [1,2,5]. Considering the cost-effectiveness of strategies for CAD prevention, incorporating lifestyle modification is still crucial for mitigating the burden of CAD [6]. Dietary adjustments, physical activity, and smoking cessation are the primary avenues through which lifestyle changes can be achieved [1,2,5].

The diet's composition impacts the body's acid-base balance [7]. Consuming a diet rich in acidic foods, including fish, cheese, meat, eggs, rice, dairy products, and cereals, while lacking in alkaline foods like fruits, vegetables, and legumes, can lead to an increase in the production of endogenous acids [8]. The imbalance in consuming more acidifying foods than alkalinizing foods is linked to lower urine pH and serum bicarbonate levels [9,10]. Increasing evidence in the literature highlights the correlation between increased dietary acidity and negative health effects, such as cardiometabolic risk factors, including hypertension, type 2 diabetes mellitus (T2DM), insulin resistance, hypertriglyceridemia, high LDL cholesterol, central obesity and kidney disease [[11], [12], [13], [14]]. The determination of dietary acid load is based on two factors, namely net endogenous acid production (NEAP) and potential renal acid load (PRAL). NEAP assesses the acidity of the diet based on the consumption of protein and potassium, while PRAL takes into account the intake of dietary calcium, phosphorus, magnesium, potassium, and protein [15]. Despite the accumulating evidence, controversies remain regarding the effects of dietary acidity measures on the development and progression of CVDs. In one study, Swedish individuals demonstrated a positive association between PRAL and CVD mortality [16]. Conversely, research on the Japanese population revealed an inverse relationship [17]. On the other hand, no significant link was found between dietary acid load and CVD incidence in the Polish population [14]. These contrasting findings underscore the complexity of the relationship between dietary acid load and cardiovascular health.

The rising popularity of acid-rich dietary patterns globally [18] poses a potential risk for the development of atherosclerotic related disorders. To the best of our knowledge, the predictive significance of dietary acid load on the risk of severe CAD progression has not been thoroughly investigated to date. In this study, we aimed to evaluate the relationship between dietary acid load, as measured by both NEAP and PRAL scores, and the progression of CAD.

## 2 Methods

### 2.1 Study population and recruitment

The current study, designed as a single-center cross-sectional analysis, utilized collected data from “The Nutrition Heshmat Registry (NUTHER)”, at Dr. Heshmat Hospital affiliated with Guilan University of Medical Sciences (GUMS), in Rasht, Iran. The collecting of data was performed between January 2022 and June 2023 (more detail is available in our previous publication from the same databank [19]). The data were collected throughout this period among subjects aged ≥20 years who met eligible criteria and admitted to the Elective Angiography Department and agreeing to join to the research. The diagnosis of CAD among participants was conducted by an expert cardiologist. The "*ESC 2019 guidelines for the diagnosis and management of chronic coronary syndromes*" [20] recommended subjects for elective angiography who experienced stable angina or atypical chest pain that may be linked to CAD. However, individuals with following criteria were excluded: those with body mass indexes (BMIs) below 18.5 or above 40 kg/m2, and those with medical histories of cardiovascular events (including myocardial infarction, percutaneous coronary intervention, or coronary artery bypass grafting). Moreover, subjects with chronic liver dysfunction, chronic kidney disease, AIDS, immune system impairments, chronic obstructive pulmonary disease, thyroid dysfunction, gout, or cancer, and those who followed special diets or did not intend to enroll in the study were additionally excluded. The NUTHER data bank ultimately compiled data from 1,235 eligible participants. Following the conclusion of data collection, individuals with more than 10 % of missing information—be it clinical or dietary—were removed from the analysis.

**Fig. 1:**
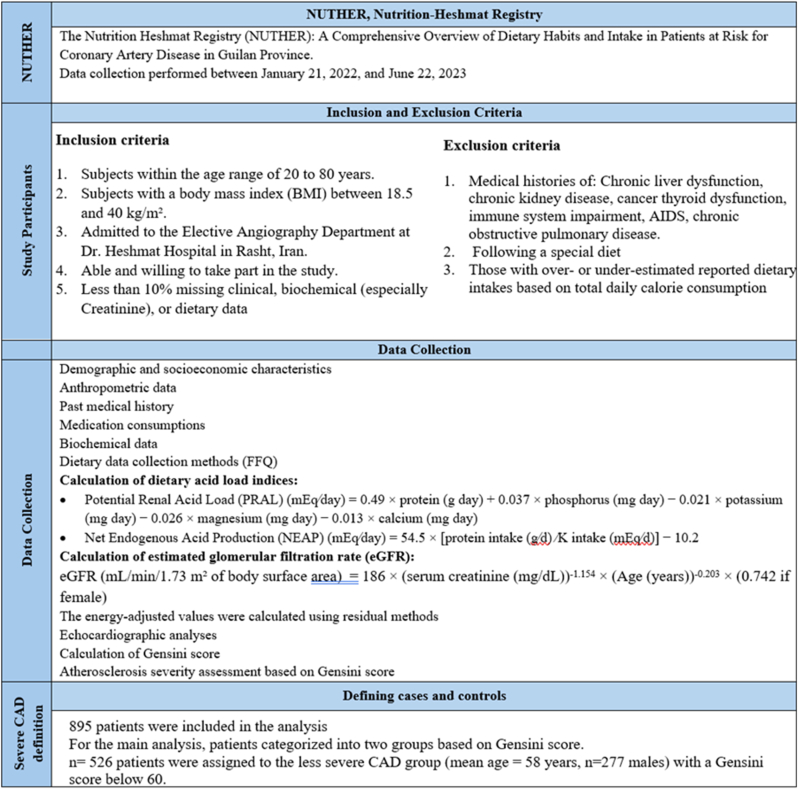
An overview of the study's design and timeline.

The research adhered to the 2013 principles outlined in the Declaration of Helsinki, and the study protocol was approved by the Institutional Review Board of the Cardiovascular Diseases Research Center at GUMS (research number 1403072309). Additionally, the GUMS Ethics Committee granted ethical approval for this study (ethics code “IR.GUMS.REC.1403.444”). Participants were made aware of the research objectives and provided their consent both in writing and verbally. An overview of the study's design and timeline is shown in Fig. 1.

### 2.2 Demographic and anthropometric data

On the day of admission, the study's aims and objectives were outlined to the participants, who then provided their signatures on informed consent forms to formally take part in the study. Data collection involved structured interviews conducted by four trained researchers. The information gathered included participants' age, educational background, gender, socioeconomic factors, demographic information, smoking habits, opioid usage, and medical history (hyperlipidemia, heart failure, family history of prediabetes/T2DM, familial history of hypertension, family history of myocardial infarction, family history of cancer, and family history of cardiovascular diseases) as well as medications consumption (anticoagulant, anti-inflammatory, anti-hyperlipidemic, anti-diabetic, and anti-hypertensive) of the subjects. Patient medical records and associated prescriptions were also reviewed to gather information on medication history. Blood pressure (systolic and diastolic) was measured with a mercury sphygmomanometer, following established and consistent procedures.

Weight and height were measured using a Seca 755 medical scale (with a precision of 0.5 kg) and a stadiometer (with an accuracy of 0.1 cm), respectively. Participants were measured while barefoot and standing relaxed with their shoulders. After obtaining these measurements, the body mass index (BMI) was calculated by dividing the weight in kilograms by the height in meters squared. Physical activity levels were assessed through a validated and reliable questionnaire and were reported in metabolic equivalent minutes per day [21].

### 2.3 Angiography of the coronary arteries, Gensini score calculation, and echocardiographic analyses

After admission, two cardiologists conducted coronary angiography using the Judkin technique via the femoral approach to assess the degree of atherosclerosis. These cardiologists, who were unaware of the study details, reviewed the angiograms and confirmed whether they showed any atherosclerotic changes in the coronary arteries. In cases of disagreement, a third cardiologist also examined the angiograms to evaluate the degree of stenosis. To assess the severity of CAD, the Gensini score, as established by Gensini et al., was utilized, focusing on the degree of stenosis in coronary lesions [22]. Initially, the most significant stenotic lesions in each artery branch were quantitatively evaluated through coronary angiography. The degrees of stenosis were classified as follows: ≤25 %, 26 %–50 %, 51 %–75 %, 76 %–90 %, 91 %–99 %, and 100 %, which corresponded to scores of 1, 2, 4, 8, 16, and 32 points, respectively. These scores were then adjusted based on the specific coronary branches according to the following multipliers: Left Main artery (LM) × 5.0, proximal Left Anterior Descending artery (LAD) × 2.5, middle LAD × 1.5, distal LAD × 1.0, proximal Left Circumflex artery (LCX) × 2.5 (3.5), middle LCX × 1.0 (2.0), distal LCX × 1.0 (2.0), Obtuse Marginal branch (OM) × 1.0, Posterior Left Ventricular artery (PLV) × 0.5, Right Coronary Artery (RCA) × 1.0, and Posterior Descending Artery (PDA) × 1.0. By adding the adjusted scores for all lesions, the total Gensini score for each individual was calculated [[23], [24], [25]]. Then after, individuals were categorized into non-severe CAD (Gensini Score<60) and severe (Gensini Score≥60) cases according to their Gensini scores.

Upon hospital admission, a certified echocardiographer measured the left ventricular ejection fraction (LVEF) as the fraction of end-diastolic blood volume ejected with each heartbeat, using a standard commercial ultrasound machine and standardized echocardiographic reports. Two cardiologists subsequently validated the calculated LVEF using the international Simpson method. Based on these results, participants were categorized into two groups: those with an LVEF of ≥50 % and those with an LVEF between 40 % and 49 %.

### 2.4 Laboratory analyses

Ten milliliters of venous blood were collected from participants who had fasted for a minimum of 8 h. To prevent clotting, the collected samples were mixed with sodium citrate in designated tubes and stored at −20 °C. Following the manufacturer's instructions, the total cholesterol levels and fasting blood glucose (FBS) were determined using the enzymatic colorimetric method. Serum triglyceride levels were measured using the enzymatic approach with commercial kits provided by Bionic Corporation (MAN Co., Tehran, Iran) and glycerol phosphate oxidase. Blood urea nitrogen was evaluated using the colorimetric method, while serum creatinine levels were analyzed according to the protocols detailed in the kit from MAN Co. in Tehran, Iran.

To estimate the estimated glomerular filtration rate (eGFR), the modification of diet in renal disease (MDRD) equation was utilized as follows [26]:

$$\text{eGFR}\;\left(\text{mL}/\min/1.73\;m^{2}\;\text{of}\;\text{body}\;\text{surface}\;\text{area}\right)=186\times {\left(\text{serum}\;\text{creatinine}\;\left(\text{mg}/\text{dL}\right)\right)}^{-1.154}\times {\left(\text{Age}\;\left(\text{years}\right)\right)}^{-0.203}\times \left(0.742\;\text{if}\;\text{female}\right)$$

### 2.5 Dietary assessments and calculating DAL

A validated 168-item semi-quantitative food frequency questionnaire (FFQ) was utilized by two trained researchers in order to collect information regarding individuals’ dietary habits. Such FFQ evaluated the regular food intake of subjects over the twelve months prior to the research, gathering information on a daily, weekly, or monthly basis [27]. The current study used the US Department of Agriculture (USDA) standard portion sizes (e.g., dairy, 1 cup; bread, 1 slice; apple, 1 medium) to estimate the serving sizes of the foods consumed. In cases where such portion sizes were not possible to apply, household measures (e.g., chicken meat, 1 leg or wing; rice, 1 large or small plate; beans, 1 tablespoon) were alternatively used [28]. Next, considering each month is 30.5 days, all the estimates were converted into grams per day and using the consumption amounts based on reported portion sizes and raw to cooked coefficients. The computing of dietary energy and nutrient composition (protein, carbohydrate, fat, animal protein, animal fat, plant protein, and plant fat) were done using USDA food composition tables (FCTs) and Nutritionist-4 software (Nutritionist IV, manufacturer). For local, culturally specific food items (for instance, mint, wild plum, sweets, and Kashk, a certain dairy products), Iranian FCTs were utilized [29,30]. To assess the energy composition and nutrient of mixed food products, including pizza, standard recipes from restaurants were utilized as references. To mitigate reporting errors, subjects who reported energy intakes above the 75th percentile or below the 25th percentile were excluded (n = 185). Therefore, males with energy intake above 3760.836 kcal/day or below 2017.344 kcal/day and females with energy intake above 3683.798 kcal/day or below 1028.198 kcal/day were excluded from the research. Two different methods, such as PRAL, and NEAP, were applied to assess the dietary acid load. These methods have been confirmed in earlier research [[31], [32], [33]].

Specifically, the formulas are as follows:

(1) PRAL (mEq/day) = (0.49 × protein (g/day)) + (0.037 × phosphorus (mg/day)) − (0.021 × potassium (mg/day)) − (0.026 × magnesium (mg/day)) − (0.013 × calcium (mg/day)) [31];
(2) NEAP (mEq/day) = (54.5 × protein intake (g/day) ÷ potassium intake (mEq/day)) − 10.2 [34];

### 2.6 Statistical methods

The energy-adjusted values of PRAL and NEAP were calculated using residual methods. The investigated continuous variables were assessed for normality using the Shapiro–Wilk test. Categorical data were presented as frequencies and percentages, with differences between quartiles assessed using chi-squared or Fisher's exact tests. Continuous variables were evaluated using linear regression to identify trends across quartiles of energy-adjusted PRAL or NEAP, with findings reported as means and standard deviations (SD) for consistency. For transparency, we also reported medians (IQR) alongside means for skewed variables in supplemental tables. Besides, sensitivity analyses were conducted using non-parametric test (e.g., Kruskal-Wallis). Crude and multiple logistic regression models were utilized to explore the relationship between severe CAD risk (defined by a Gensini score ≥60) and quartiles of energy-adjusted PRAL and NEAP. The first multiple regression models were adjusted for age and daily energy intake, while subsequent models incorporated additional covariates such as BMI (kg/m^2^) eGFR (mL/min/1.73 m^2^), gender, smoking, opium use, education, LVEF category, hypertension, prediabetes/T2DM, dyslipidemia, anticoagulant use, anti-inflammatory drugs use, anti-hyperlipidemic use, antidiabetics use, and physical activity (measured in metabolic equivalent minutes per day). Odds ratios (OR) with 95 % confidence intervals (CI) were computed. To evaluate linear trends across quartiles, the median values of each quartile were treated as continuous variables, and P-for-trend was calculated. We also performed additional sensitivity analyses using alternative cut-offs for Gensini scores (≥40 vs. <40 and ≥ 50 vs. <50) to assess the robustness of our findings.

To explore potential nonlinear associations between PRAL, and NEAP and severe CAD risk, restricted cubic spline (RCS) regression with three knots (positioned at the 5th, 50th, and 95th percentiles of the exposure variables) was employed, adjusting for relevant covariates. All statistical analyses were conducted using STATA version 17 (StataCorp LLC, College Station, TX, USA).

## 3 Results

### 3.1 Study participants characteristics

Following the exclusion of individuals with over 10 % missing clinical or dietary information, as well as those with over- or under-estimated reported dietary intakes based on total daily calorie consumption, 895 patients were analyzed. Of these, 526 patients were assigned to the less severe CAD group (mean age = 58 years, n = 277 males) with a Gensini score below 60, while 369 patients were placed in the severe CAD group (mean age = 59 years, n = 222 males) with a Gensini score of 60 or higher (Fig. 1).

Table 1 presents the demographic characteristics of study participants stratified by energy-adjusted PRAL and NEAP quartiles (mEq∕day). The data reveal that participants in the top quartile of both PRAL and NEAP scores were more likely to exhibit higher Gensini scores and slightly lower BMI compared to those in the bottom quartile. Furthermore, individuals in the highest quartiles of NEAP and PRAL scores had lower LVEF than those in the lowest quartiles (P-value<0.05). No significant associations were found between different levels of quartiles of PRAL and NEAP and gender, marital status, education, smoking habits, opiate use, physical activity levels, prevalence of chronic illnesses, family history of chronic illnesses, biochemical measurements, or the use of medications. Besides, sensitivity analyses for continuous variable were conducted using non-parametric test (e.g., Kruskal-Wallis) confirmed consistent results (Supplementary Tables S1–S5).

**Table 1:** Studied participants characteristics across quartiles of dietary acid load indices in a cross-sectional study on coronary artery disease patients.

	Quartiles of energy-adjusted Potential Renal Acid Load (PRAL)				*P value*^*†*^	Quartiles of Net Endogenous Acid Production (NEAP)				*P value*^*†*^
Variable	Q1	Q2	Q3	Q4		Q1	Q2	Q3	Q4	
	N = 224	N = 224	N = 224	N = 223		N = 224	N = 224	N = 224	N = 223	
**Demographic data**										
Age (y)	58.57 (11.08)	57.62 (10.55)	58.90 (10.23)	58.43 (10.57)	0.828	58.50 (11.11)	57.92 (10.70)	58.32 (10.16)	58.78 (10.49)	0.654
Gender (male, n (%))	130 (58.0 %)	125 (55.8 %)	117 (52.2 %)	127 (57.0 %)	0.634	130 (58.0 %)	121 (54.0 %)	116 (51.8 %)	132 (59.2 %)	0.359
Married (n (%))	195 (87.1 %)	199 (88.8 %)	209 (93.3 %)	200 (89.7 %)	0.145	192 (85.7 %)	201 (89.7 %)	207 (92.4 %)	203 (91.0 %)	0.122
Education					0.928					0.984
Illiterate or elementary school (n (%))	125 (55.80 %)	130 (58.04 %)	126 (56.25 %)	128 (57.40 %)		131 (58.48 %)	127 (56.70 %)	126 (56.25 %)	125 (56.05 %)	
Middle school (n (%))	76 (33.93 %)	63 (28.13 %)	66 (29.46 %)	68 (30.49 %)		71 (31.70 %)	66 (29.46 %)	68 (30.36 %)	68 (30.49 %)	
Diploma and Higher (n (%))	23 (10.27 %)	31 (13.79 %)	32 (14.29 %)	27 (12.14 %)		22 (9.82 %)	31 (13.84 %)	30 (13.39 %)	30 (13.95 %)	
Smoking (n (%))	45 (20.1 %)	49 (21.9 %)	36 (16.1 %)	57 (25.6 %)	0.097	44 (19.6 %)	45 (20.1 %)	40 (17.9 %)	58 (26.0 %)	0.178
Opium (n (%))	34 (15.2 %)	44 (19.6 %)	38 (17.0 %)	37 (16.6 %)	0.661	31 (13.8 %)	48 (21.4 %)	35 (15.6 %)	39 (17.5 %)	0.183
Physical activity (metabolic equivalent minutes/day)	32.88 (27.19)	26.88 (27.12)	32.75 (28.35)	32.23 (27.00)	0.727	32.52 (27.23)	28.66 (27.94)	31.79 (27.66)	31.75 (27.14)	0.914
Past medical history										
Hypertension (n (%))	167 (74.6 %)	156 (69.6 %)	154 (68.8 %)	161 (72.2 %)	0.524	167 (74.6 %)	155 (69.2 %)	154 (68.8 %)	162 (72.6 %)	0.467
Dyslipidemia (n (%))	183 (81.7 %)	183 (81.7 %)	189 (84.4 %)	174 (78.0 %)	0.403	172 (76.8 %)	192 (85.7 %)	180 (80.4 %)	185 (83.0 %)	0.094
Prediabetes/diabetes (n (%))	159 (71.0 %)	155 (69.2 %)	151 (67.4 %)	152 (68.2 %)	0.858	157 (70.1 %)	155 (69.2 %)	152 (67.9 %)	153 (68.6 %)	0.96
FHDM (n (%))	53 (23.7 %)	64 (28.6 %)	62 (27.7 %)	67 (30.0 %)	0.469	51 (22.8 %)	63 (28.1 %)	65 (29.0 %)	67 (30.0 %)	0.312
FHHTN (n (%))	69 (30.8 %)	58 (25.9 %)	72 (32.1 %)	65 (29.1 %)	0.504	64 (28.6 %)	65 (29.0 %)	65 (29.0 %)	70 (31.4 %)	0.914
FHMI (n (%))	40 (17.9 %)	39 (17.4 %)	53 (23.7 %)	41 (18.4 %)	0.325	42 (18.8 %)	36 (16.1 %)	47 (21.0 %)	48 (21.5 %)	0.452
FH cancer (n (%))	22 (9.8 %)	17 (7.6 %)	20 (8.9 %)	17 (7.6 %)	0.812	21 (9.4 %)	16 (7.1 %)	21 (9.4 %)	18 (8.1 %)	0.789
FHCVDs (n (%))	95 (42.4 %)	99 (44.2 %)	100 (44.6 %)	104 (46.6 %)	0.853	96 (42.9 %)	96 (42.9 %)	96 (42.9 %)	110 (49.3 %)	0.421
Medication use										
Anti-inflammatory drugs (n (%))	187 (83.5 %)	182 (81.3 %)	179 (79.9 %)	172 (77.1 %)	0.389	187 (83.5 %)	182 (81.3 %)	178 (79.5 %)	173 (77.6 %)	0.443
Anticoagulant drugs (n (%))	18 (8.0 %)	20 (8.9 %)	17 (7.6 %)	22 (9.9 %)	0.834	16 (7.1 %)	22 (9.8 %)	16 (7.1 %)	23 (10.3 %)	0.487
Anti-hyperlipidemic (n (%))	135 (60.3 %)	130 (58.0 %)	129 (57.6 %)	127 (57.0 %)	0.903	134 (59.8 %)	127 (56.7 %)	123 (54.9 %)	137 (61.4 %)	0.487
Antidiabetics (n (%))	113 (50.4 %)	119 (53.1 %)	108 (48.2 %)	112 (50.2 %)	0.782	113 (50.4 %)	115 (51.3 %)	115 (51.3 %)	109 (48.9 %)	0.945
Anti-hypertensive (n (%))	128 (57.1 %)	126 (56.3 %)	115 (51.3 %)	126 (56.5 %)	0.586	127 (56.7 %)	126 (56.3 %)	115 (51.3 %)	127 (57.0 %)	0.592
Anthropometric and biochemical data										
BMI (kg/m^2^)	28.05 (4.74)	27.85 (4.98)	27.45 (4.61)	27.23 (4.03)	0.040	28.50 (5.12)	27.62 (4.51)	27.22 (4.70)	27.25 (3.93)	0.004
Fasting blood sugar (mg/dL)	141.51 (74.69)	140.19 (64.18)	134.00 (69.50)	141.00 (70.00)	0.710	137.39 (71.35)	138.44 (62.03)	141.88 (76.78)	138.98 (67.95)	0.751
Cholesterol (mg/dL)	164.47 (50.11)	161.58 (52.45)	157.66 (44.49)	160.65 (42.48)	0.274	165.19 (49.09)	158.15 (45.63)	162.79 (51.75)	158.22 (43.17)	0.227
Triglyceride (mg/dL)	164.96 (94.94)	172.83 (110.21)	166.49 (99.41)	168.25 (121.15)	0.882	163.51 (98.21)	168.75 (90.92)	169.69 (113.21)	170.59 (122.48)	0.504
eGFR (mL/min/1.73 m^2^)	74.53 (21.75)	71.86 (19.79)	73.96 (20.05)	75.23 (19.88)	0.544	74.29 (22.27)	72.16 (18.55)	74.13 (19.95)	74.99 (20.65)	0.480
Creatinine (mg/dL)	0.98 (0.23)	1.00 (0.21)	0.96 (0.22)	0.96 (0.22)	0.199	0.99 (0.24)	0.99 (0.20)	0.96 (0.21)	0.97 (0.23)	0.384
Blood urea nitrogen (BUN) (mg/dL)	15.58 (4.19)	16.61 (4.62)	15.97 (4.11)	15.26 (3.56)	0.253	15.70 (4.42)	16.34 (4.11)	15.88 (4.29)	15.50 (3.79)	0.328
Energy-adjusted PRAL (mEq∕day)	−10.75 (9.44)	1.05 (4.04)	8.59 (4.07)	17.32 (5.39)	<0.001	−10.75 (9.44)	1.05 (4.04)	8.59 (4.07)	17.32 (5.39)	<0.001
Energy-adjusted NEAP (mEq∕day)	42.31 (5.86)	52.19 (4.06)	60.68 (5.72)	74.23 (8.98)	<0.001	41.61 (5.30)	52.03 (2.11)	60.16 (3.06)	75.62 (7.30)	<0.001
Angiographic and echocardiographic data										
LVEF category										
40–49	75 (33.5 %)	89 (39.7 %)	68 (30.4 %)	109 (48.9 %)	<0.001	74 (33.0 %)	82 (36.6 %)	74 (33.0 %)	111 (49.8 %)	<0.001
≥50	149 (66.5 %)	135 (60.3 %)	156 (69.6 %)	114 (51.1 %)		150 (67.0 %)	142 (63.4 %)	150 (67.0 %)	112 (50.2 %)	
Gensini Score	46.84 (39.81)	55.73 (39.91)	55.53 (39.62)	63.16 (38.28)	<0.001	43.02 (38.15)	54.44 (40.37)	59.24 (41.37)	64.57 (35.99)	<0.001

∗All values are mean ± SD, unless indicated; †Linear regression for continuous variables and Chi-squared test for categorical variables. BMI: body mass index; FBS: fasting blood sugar; HDL: high-density lipoprotein; LDL: low-density lipoprotein; HLP: hyperlipidemia; HF: heart failure; FHDM: family history of diabetes; FHHTN: familial history of hypertension; FHMI: family history of myocardial infarction; FH cancer: family history of cancer; FHCVDs: family history of cardiovascular diseases; LVEF: left ventricular ejection fraction; eGFR: estimated glomerular filtration rate.

Table 2 compares participants' energy intake from macro- and micronutrients across quartiles of energy-adjusted PRAL and NEAP scores (mEq∕day). Subjects in quartile 4 of both NEAP and PRAL scores were more likely to consume higher amounts of total protein, saturated fatty acids, meat, and grains per day compared to those in quartile 1. Conversely, their intake of magnesium, phosphorus, potassium, vegetables, and fruits was significantly lower (P-value<0.05).

**Table 2:** Studied participants dietary intakes across quartiles of dietary acid load indices in a cross-sectional study on coronary artery disease patients.

	Quartiles of energy-adjusted Potential Renal Acid Load (PRAL)				*P value*^*†*^	Quartiles of Net Endogenous Acid Production (NEAP)				*P value*^*†*^
Variable	Q1	Q2	Q3	Q4		Q1	Q2	Q3	Q4	
	N = 224	N = 224	N = 224	N = 223		N = 224	N = 224	N = 224	N = 223	
Energy (Kcal/day)	3368.37 (737.86)	3150.66 (691.25)	3104.39 (662.66)	3439.53 (699.34)	0.535	3369.39 (810.30)	3163.30 (663.88)	3072.09 (590.29)	3458.26 (699.19)	0.119
Protein (g/day)	99.27 (20.98)	95.93 (21.47)	95.37 (20.51)	108.66 (22.76)	<0.001	98.54 (23.36)	96.72 (21.40)	95.94 (19.54)	108.04 (21.76)	<0.001
Carbohydrate (g/day)	477.84 (130.51)	432.31 (119.28)	421.63 (117.71)	478.05 (126.88)	0.658	476.04 (140.49)	432.67 (114.87)	412.84 (105.21)	488.32 (126.72)	0.199
Fat (g/day)	111.79 (28.07)	107.42 (25.81)	106.64 (24.08)	112.39 (24.63)	0.966	113.72 (30.26)	108.63 (24.73)	106.25 (22.73)	109.64 (24.35)	0.108
Saturated fatty acid (g/day)	47.25 (13.35)	46.69 (12.07)	46.61 (11.73)	50.43 (11.21)	0.011	47.61 (14.32)	47.42 (11.70)	46.01 (10.83)	49.93 (11.39)	0.056
Magnesium (mg/day)	357.06 (81.12)	305.01 (69.20)	285.79 (65.29)	275.53 (66.57)	<0.001	367.34 (89.08)	308.72 (64.85)	287.00 (51.65)	260.27 (54.43)	<0.001
Phosphorus (mg/day)	1283.42 (296.69)	1178.33 (265.39)	1139.94 (257.66)	1185.76 (276.22)	<0.001	1330.87 (344.11)	1197.14 (252.14)	1148.30 (214.52)	1110.83 (237.68)	<0.001
Potassium (mg/day)	3975.55 (738.44)	3292.18 (562.61)	2901.94 (527.49)	2713.65 (561.23)	<0.001	3987.45 (790.94)	3317.71 (550.60)	2941.20 (478.22)	2636.60 (462.80)	<0.001
calcium (mg/day)	1105.49 (303.90)	1014.15 (263.51)	991.55 (260.05)	1069.33 (290.63)	0.092	1112.66 (316.93)	1041.10 (278.78)	982.06 (245.84)	1044.59 (273.38)	0.008
Meat (g/day)	96.89 (32.55)	104.06 (33.10)	103.02 (31.91)	122.96 (41.30)	<0.001	93.30 (32.22)	105.15 (33.53)	110.59 (36.36)	117.87 (38.12)	<0.001
Egg (g/day)	12.70 (10.21)	13.55 (10.70)	12.96 (10.81)	15.13 (11.51)	0.038	12.38 (10.35)	14.12 (11.00)	13.71 (12.16)	14.11 (9.67)	0.149
Vegetables (g/day)	369.92 (143.71)	303.62 (100.02)	260.91 (78.65)	236.04 (82.59)	0.912	361.33 (145.80)	305.65 (102.41)	269.34 (83.31)	234.17 (80.24)	<0.001
Fruits (g/day)	518.84 (207.51)	362.66 (146.06)	280.26 (104.50)	233.88 (108.94)	0.912	494.16 (219.50)	373.62 (152.65)	286.24 (125.01)	241.66 (99.84)	<0.001
Grains (g/day)	682.79 (251.59)	663.12 (236.39)	713.01 (244.48)	837.05 (279.54)	<0.001	692.21 (271.60)	677.02 (244.90)	686.14 (211.45)	840.62 (280.53)	<0.001
Dairy (g/day)	441.86 (214.87)	413.93 (176.86)	410.04 (181.00)	446.76 (200.72)	0.912	437.33 (216.31)	440.65 (192.01)	406.86 (173.45)	427.66 (192.65)	0.375

∗All values are mean ± SD; †Linear regression.

### 3.2 The regression analysis of the association between dietary acid load indices in relation to severe CAD risk

Table 3 presents the crude and multivariable-adjusted ORs and 95 % CIs for severe CAD across quartiles of energy-adjusted dietary acid load measures (PRAL and NEAP). The results indicate that the odds of severe CAD consistently increased with higher quartiles of both PRAL and NEAP across all models.

**Table 3:** Odds Ratios (ORs) and 95 % Confidence Interval (95 %CI) of severe coronary artery disease according to quartiles of dietary acid load indices.

Variable	Quartiles of Dietary acid load				P for trend
	1st	2nd	3rd	4th	
**Quartilesofenergy-adjustedPotentialRenalAcidLoad(PRAL)(mEq∕day)**					
**Median**	−8.67	0.95	8.51	16.91	
**non-cases/Cases**	151/73	128/96	130/94	117/106	
**Crude model**	1.00 (Ref)	1.55 (1.06, 2.28)	1.50 (1.02, 2.20)	1.87 (1.28, 2.75)	0.002
**Model ^a^**	1.00 (Ref)	1.70 (1.15, 2.52)	1.64 (1.10, 2.43)	1.86 (1.26, 2.73)	0.003
**Model ^b^**	1.00 (Ref)	1.57 (1.01, 2.45)	1.80 (1.16, 2.79)	1.62 (1.05, 2.51)	0.021
**Quartiles of energy-adjustedNet Endogenous Acid Production (NEAP)(mEq∕day)**					
**Median**	42.79	52.25	60.11	74.18	
**non-cases/Cases**	157/67	135/89	117/107	117/106	
**Crude model**	1.00 (Ref)	1.54 (1.04, 2.29)	2.14 (1.45, 3.16)	2.12 (1.44, 3.13)	<0.001
**Model ^a^**	1.00 (Ref)	1.70 (1.14, 2.53)	2.44 (1.63, 3.63)	2.09 (1.41, 3.09)	<0.001
**Model ^b^**	1.00 (Ref)	1.54 (0.98, 2.42)	2.76 (1.76, 4.33)	1.67 (1.07, 2.61)	0.012

• Model ^a^: Adjusted for age and total energy intake.

• Model ^b^: Further adjusted for BMI (kg/m^2^) eGFR (mL/min/1.73 m^2^), gender, smoking, opium use, education, LVEF category, hypertension, prediabetes/T2DM, dyslipidemia, anticoagulant use, anti-inflammatory drugs use, anti-hyperlipidemic use, antidiabetics use, and physical activity.

• Ref: Reference category.

For PRAL, in the crude model, the ORs for the 2nd (median = 0.95 mEq∕day), 3rd (median = 8.51 mEq∕day), and 4th (median = 16.91 mEq∕day) quartiles were 1.55, 1.50, and 1.87, respectively, compared to the 1st quartile (median = −8.67 mEq∕day). After adjusting for age and total energy intake in Model a, the ORs increased slightly to 1.70, 1.64, and 1.86 for the 2nd, 3rd, and 4th quartiles, respectively. In the fully adjusted model (Model b), which included additional adjustments for BMI (kg/m^2^) eGFR (mL/min/1.73 m^2^), gender, smoking, opium use, education, LVEF category, hypertension, prediabetes/T2DM, dyslipidemia, anticoagulant use, anti-inflammatory drugs use, anti-hyperlipidemic use, antidiabetics use, and physical activity, the odds of severe CAD increased by 1.57- to 1.8-fold across the PRAL quartiles. The fully adjusted ORs (95 % CIs) were: Q2: 1.57 (1.01, 2.45); Q3: 1.80 (1.16, 2.79); and Q4: 1.62 (1.05, 2.51); (P for trend = 0.021), indicating a consistent association between higher PRAL levels and increased odds of severe CAD.

Similarly, the odds of severe CAD increased with higher quartiles of NEAP in all models. In the crude model, the ORs for the 2nd (median = 52.25 mEq∕day), 3rd (median = 60.11 mEq∕day), and 4th (median = 74.18 mEq∕day) quartiles were 1.54, 2.14, and 2.12, respectively, compared to the 1st quartile (median = 42.79 mEq∕day). After adjusting for age and total energy intake (Model a), the ORs increased to 1.70, 2.44, and 2.09 for the 2nd, 3rd, and 4th quartiles, respectively. In the fully adjusted model (Model b), higher NEAP levels were associated with increased odds of severe CAD, though this association was significant only for the 3rd and 4th quartiles, with ORs (95 % CIs) of 2.76 (1.76, 4.33) and 1.67 (1.07, 2.61), respectively (P for trend = 0.012)

To assess the robustness of our findings, further sensitivity analyses using alternative cut-offs for Gensini scores (≥40 vs. <40 and ≥ 50 vs. <50) were conducted and consistently confirm the current findings of an increased risk of severe CAD in association with higher dietary acid load measures (Supplementary Tables S5–S6).

### 3.3 Dose-response relationship analysis of the association between dietary acid load indices in relation to severe CAD risk

**Fig. 2:**
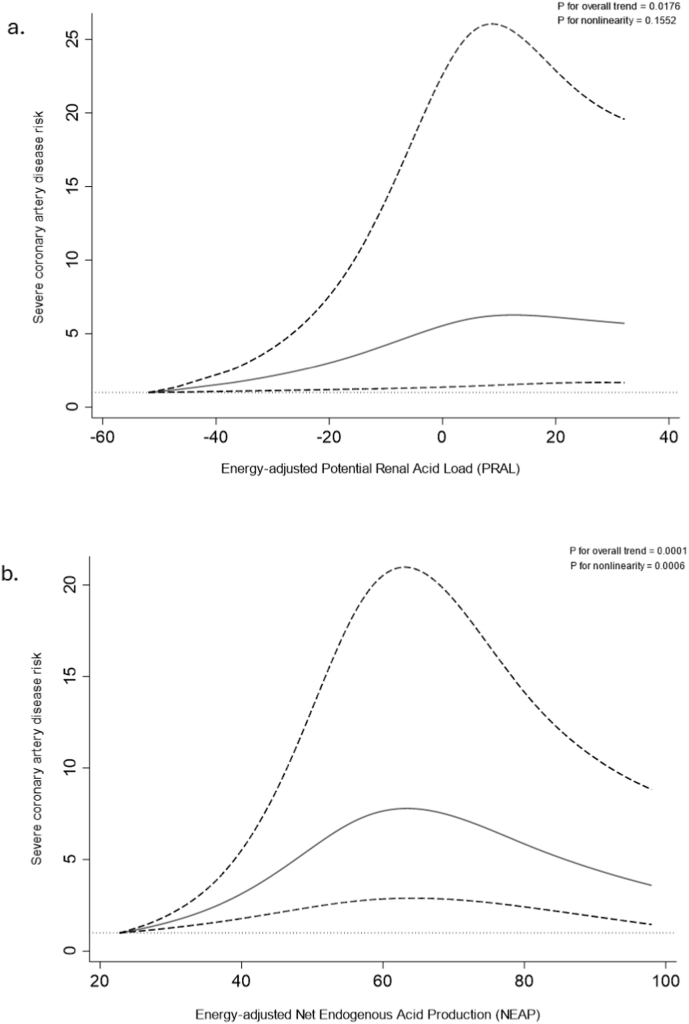
Restricted cubic spline regression plots demonstrating the relationship between dietary acid load indices, energy-adjusted Potential Renal Acid Load (PRAL) (a.) and Net Endogenous Acid Production (NEAP) Net Endogenous Acid Production (NEAP) (b.), with the risk of severe coronary artery disease (CAD). • Fig. 2a: This model examines the link between the **energy-adjusted Potential Renal Acid Load (PRAL)** and severe CAD risk, adjusted for various factors such as age and total energy intake, BMI (kg/m^2^), eGFR (mL/min/1.73 m^2^). gender, smoking, opium use, education, LVEF category, hypertension, prediabetes/T2DM, dyslipidemia, anticoagulant use, anti-inflammatory drugs use, anti-hyperlipidemic use, antidiabetics use, and physical activity. The model uses cubic knots at the 5th **(-17.27**), 50th (**4.72**), and 95th (**22.80**) percentiles. • Fig. 2b: This model evaluates the association between **energy-adjusted Net Endogenous Acid Production (NEAP)** and severe CAD risk, adjusted for the same variables as Fig. 2a. Cubic knots were placed at the 5th (**37.79**), 50th (**55.40**), and 95th (**81.71**) percentiles. In all plots, the solid lines represent odds ratios (ORs), and the shaded regions show the 95 % confidence intervals (95 % CI).

Fig. 2 presents RCS regression plots demonstrating the relationship between dietary acid load measures, energy-adjusted PRAL (Fig. 2 a.) and NEAP (Fig. 2 b.), with the risk of severe CAD. Three knots placed at the 5th, 50th, and 95th percentiles of each variable. For energy-adjusted PRAL, a linear dose-response relationship was found with severe CAD risk (P for overall trend = 0.0176; P for nonlinearity = 0.1552), as shown in Fig. 2a. While NEAP exerted a more nonlinear association with higher severe CAD risk (P for overall trend = 0.0001; P for nonlinearity = 0.006), as shown in Fig. 2b).

## 4 Discussion

This cross-sectional study, involving 895 895 Iranian adults with moderate BMI referred to the elective angiographic department and categorized based on Gensini score severity (526 in the non-severe CAD group and 369 in the severe CAD group), revealed a significant increase in severe CAD risk associated with higher dietary acid load as measured by energy-adjusted PRAL and NEAP, even after controlling for several confounding variables, including demographic, socioeconomic, and clinical factors (such as chronic disorder histories and medication use), physical activity level, and daily energy intake. Specifically, there was an increase in severe CAD risk of 57–80 % across the 2nd to 4th quartiles of PRAL and 67–176 % across the 3rd to 4th quartiles of NEAP. Interestingly, higher dietary acid load indices in the current study were accompanied by greater consumption of total protein, saturated fatty acids, meat, and grains, alongside lower consumption of magnesium, phosphorus, potassium, vegetables, and fruits in this study. This dietary pattern could potentially contribute to an imbalance in the body's acid-base homeostasis, leading to metabolic acidosis. Furthermore, the dose-response relationship between dietary acid load measures and the risk of severe CAD varies between energy-adjusted PRAL and NEAP. The RCS analysis shows a linear dose-response for energy-adjusted PRAL concerning severe CAD risk, while NEAP exhibits a nonlinear relationship with higher risk of severe CAD. This difference may arise from how PRAL and NEAP are calculated. PRAL involves a more complex formula that takes into account key dietary elements like magnesium, calcium, and phosphorus, whereas NEAP relies on a simpler calculation that only considers protein and potassium intake. Therefore, it's possible that the inclusion of magnesium, calcium, and phosphorus in the PRAL formula influences its relationship with severe CAD risk, especially given the ongoing research into how these factors relate to cardiovascular diseases. In contrast, NEAP's focus solely on potassium and protein may overlook critical dietary factors contributing to CAD risk.

Similar to our obtained results, a cross-sectional study conducted in 2016 utilizing the Korea National Health and Nutrition Examination data highlighted a direct relationship between dietary acid load and cardiovascular disease risk [35]. Additionally, findings from a meta-analysis of 55 observational studies in 2024 reported a significant association between dietary acid loads and development of cardiometabolic risk factors such as blood pressure, anthropometric indices, and triglyceride [36]. Several published studies have investigated the relationship between dietary acid load and the risk of various CVD risk factors. These studies have consistently demonstrated a direct association between higher dietary acid load and an increased risk of developing CVD-related risk factors [[37], [38], [39], [40]]. For instance, NEAP showed a non-linear association with hypertension, while every 20-unit rise in PRAL was related to a 3 % elevation in the odds of hypertension in a meta-analysis conducted in 2018 [38]. However, in contrast to our findings, a previously published prospective study involving Asian subjects with approximately 7 years of follow-up did not find a significant relationship between dietary acid load and an increased risk of CVDs [41]. It seems that such a result might stem from limited study participants, a short follow-up period, the young age of individuals, as well as alterations in dietary patterns over time. Also, a comprehensive study conducted in Poland could not find a relationship between DAL and CVD [42]. The observed variations between the present cross-sectional research and these studies could be explained by differences in baseline population characteristics and usual dietary intake, which impact the spectrum of dietary acid load. In this regard, further prospective studies are recommended to shed light on this relationship.

A wide range of physiological mechanisms may explain the relationship between dietary acid load and CAD progression. A high-acid diet, characterized by greater intake of animal proteins and lower consumption of alkaline foods such as fruits and vegetables (as also observed in our study population), can lead to low-grade acidosis. This condition has been linked to metabolic complications, namely hypertension, diabetes, and renal and bone-related disorders [43,44]. Strong adherence to the Western diet is known to increase metabolic acidosis, which is linked to the rise in blood pressure through several mechanisms such as lower citrate excretion, elevated cortisol secretion, and calcium retention. Furthermore, higher secretion of cortisol may play a role in exacerbating other metabolic risk factors [31,45,46]. Additionally, increased dietary acid load negatively impacts parathyroid function and broader endocrine systems, contributing to "metabolic-endocrine stress." Clinical signs include insulin sensitivity, and markers of bone loss [43]. A potassium-restricted diet can impair the function of blood vessels and vascular dilation and cause potassium deficiencies. Such deficiency can stimulate sodium accumulation in order to maintain the stability of a cell's tonicity and volume [45]. Excessive consumption of diets with high acid load might also be associated with reduced kidney function and metabolic performance, insulin resistance, as well as increased incidence of peripheral artery disease and T2DM [[47], [48], [49]]. Animal model studies have demonstrated that metabolic acidosis accounts for impairing the binding of insulin to its receptors, which highlighted the hypothesis regarding metabolic acidosis may have a potential to promote insulin resistance [50]. Such an insulin resistance leads to hyperglycemia and consequently increased the likelihood of CVDs and CAD progression [51,52].

### 4.1 Study strengths and limitations

This research employs a cross-sectional design to unveil significant strengths and some limitations. The nutritional intakes of the participants, that were collected by trained personnel using a validated 168-item FFQ, were used to calculate the dietary acid load. The severity of CAD was accurately measured through the Gensini scoring system, based on precise coronary angiography examinations, a verified approach performed by two interventional cardiologists. Furthermore, multiple regression RCS analyses were conducted, taking into account various potential covariate factors, to investigate the dose-response relationship between dietary acid load measures and the risk of severe CAD.

On the other hand, when interpreting the results of this study, several limitations must be considered. Despite using a validated FFQ, recall bias might influence the outcomes. Furthermore, due to the study's restriction on Iranian individuals and exclusion of participants with BMI less than 18.5 or more than 40 kg/m^2^, the results may not be applicable to various populations, especially those with different dietary patterns such as the Mediterranean diet or higher obesity rates. Additional studies in broader populations are necessary to confirm such results. Additionally, the single-center and cross-sectional design of the study limits the ability to establish cause-and-effect relationships between dietary acid load measures, and coronary artery disease. To validate these associations, further prospective studies and well-designed randomized controlled trials are needed. Eventually, limited information on markers of inflammation and oxidative stress, along with the lack of analysis relating dietary acid load to lipid profiles, may have led to an inaccurate estimation of the strength of the association. Therefore, additional experimental research is needed to explore the underlying mechanisms of this relationship.

#### 4.1.1 Clinical relevance of the study findings

The current study's findings suggest that a higher dietary acid load, which appears to contribute to an imbalance in the body's acid-base homeostasis, is characterized by increased consumption of total protein, saturated fatty acids, meat, and grains, alongside reduced intake of magnesium, phosphorus, potassium, vegetables, and fruits. These results highlight the importance of dietary interventions aimed at lowering dietary acid load to reduce the risk of CAD progression. Clinicians should consider advising patients, especially those at high risk for CAD, to adopt a diet rich in alkaline foods such as fruits and vegetables, while limiting the intake of acidic foods like meat, saturated fats, and grains. Furthermore, monitoring and managing patients' dietary intakes using indices like PRAL and NEAP could be a practical strategy for the prevention and management of CAD.

## 5 Conclusion

This cross-sectional study involving 895 Iranian adults with moderate BMI, categorized by Gensini score severity (526 participants in the non-severe CAD group and 369 in the severe CAD group), found a significant association between higher dietary acid load—measured by energy-adjusted PRAL and NEAP—and increased severe CAD risk. Specifically, there was a 57–80 % increase in severe CAD risk across the 2nd to 4th quartiles of PRAL and a 67–176 % increase across the 3rd to 4th quartiles of NEAP. Moreover, RCS analysis revealed linear dose-response relationships between elevated PRAL and severe CAD risk, along with a nonlinear association between higher NEAP and increased severe CAD risk. However, such findings should be interpreted cautiously among populations who follow different dietary patterns or having a wide range of BMI.

Considering the growing global prevalence of CADs, the heavy treatment costs, and the significant burden on healthcare systems, as well as the main role of diet on cardiovascular health, additional investigations in other regions and populations with larger sample sizes and extended follow-up periods are required to know how dietary acid load influences CAD development.

## CRediT authorship contribution statement

**Zeinab Ghorbani:** Writing – review & editing, Writing – original draft, Visualization, Validation, Supervision, Software, Resources, Investigation, Funding acquisition, Formal analysis, Data curation, Conceptualization. **Fatemeh Dashti:** Writing – review & editing, Writing – original draft, Resources, Investigation, Data curation. **Zahra Saeedirad:** Writing – review & editing, Writing – original draft, Resources, Investigation, Data curation. **Amir Aris:** Writing – review & editing, Resources, Methodology, Investigation, Data curation. **Marjan Mahdavi-Roshan:** Writing – review & editing, Writing – original draft, Validation, Supervision, Resources, Project administration, Methodology, Data curation, Conceptualization. **Arsalan Salari:** Writing – review & editing, Validation, Resources, Investigation, Data curation.

## Ethical approval and consent

The research adhered to the 2013 principles outlined in the Declaration of Helsinki, and the study protocol was approved by the Institutional Review Board of the Cardiovascular Diseases Research Center at GUMS (research number 1403072309). Additionally, the GUMS Ethics Committee granted ethical approval for this study (ethics code “IR.GUMS.REC.1403.444”).

## Consent for publication

All authors have read and consented to the publication of this manuscript.

## Availability of data and materials

The datasets of the current study are available from the corresponding author on reasonable request.

## Funding

This study was financially supported by the Cardiovascular Diseases Research Center, GUMS (research code=1403072309).
