# Supplementary material for: Increased dietary acid load May elevate the risk of coronary artery disease severity: Findings from a cross-sectional study
Source: Int J Cardiol Cardiovasc Risk Prev. 2025 May 15;26:200423. doi: 10.1016/j.ijcrp.2025.200423 (PMC12148600; doi:10.1016/j.ijcrp.2025.200423)
Supplement: Multimedia component 1 [file mmc1.docx]

| **Table S1: Median (IQR) values of age, physical activity, CAD severity (Gensini score), metabolic, and clinical parameters, across quartiles of energy-adjusted Potential Renal Acid Load (PRAL) in a cross-sectional study.** | | | | | | |
| --- | --- | --- | --- | --- | --- | --- |
|  | **Quartiles of PRAL** | | | | **Test** | **p-value** |
|  | **Q1** | **Q2** | **Q3** | **Q4** |  |  |
|  | **N=224** | **N=224** | **N=224** | **N=223** |  |  |
| **Age (y)** | 58.00 (51.00-67.00) | 58.00 (50.00-65.00) | 58.00 (52.00-66.00) | 59.00 (51.00-66.00) | Kruskal-Wallis | 0.62 |
| **Physical activity (metabolic equivalent minutes/day)** | 30.00 (10.00-50.00) | 15.00 (5.00-45.00) | 25.00 (10.00-50.00) | 30.00 (10.00-50.00) | Kruskal-Wallis | 0.027 |
| **Gensini Score** | 33.50 (11.00-79.25) | 49.25 (17.00-84.75) | 49.50 (17.25-86.50) | 53.50 (38.00-84.50) | Kruskal-Wallis | <0.001 |
| **BMI (kg/m^2^)** | 27.54 (24.66-31.25) | 26.90 (24.61-30.84) | 26.91 (24.35-29.83) | 26.57 (24.57-29.68) | Kruskal-Wallis | 0.26 |
| **Fasting blood sugar (mg/dL)** | 114.00 (98.00-153.00) | 117.00 (99.00-158.50) | 108.00 (98.00-144.00) | 115.00 (99.00-151.00) | Kruskal-Wallis | 0.20 |
| **Cholesterol (mg/dL)** | 156.50 (131.50-194.00) | 150.50 (129.50-182.00) | 149.00 (126.00-182.00) | 152.00 (133.00-184.00) | Kruskal-Wallis | 0.44 |
| **Triglyceride (mg/dL)** | 142.50 (104.50-197.50) | 142.00 (104.00-207.50) | 142.00 (103.50-198.00) | 141.00 (104.00-192.00) | Kruskal-Wallis | 0.92 |
| **eGFR (mL/min/1.73 m²)** | 73.59 (58.36-86.91) | 69.06 (57.72-84.12) | 70.76 (59.30-87.03) | 73.92 (61.07-86.16) | Kruskal-Wallis | 0.41 |
| **Creatinine (mg/dL)** | 0.94 (0.80-1.13) | 0.96 (0.87-1.12) | 0.91 (0.81-1.10) | 0.95 (0.80-1.08) | Kruskal-Wallis | 0.14 |
| **Blood urea nitrogen (BUN) (mg/dL)** | 15.00 (13.00-17.00) | 16.00 (13.00-19.00) | 15.00 (13.00-18.00) | 15.00 (13.00-17.00) | Kruskal-Wallis | 0.012 |
| **Energy-adjusted PRAL (mEq∕day)** | -9.35 (-15.00--5.02) | -0.08 (-2.21-2.59) | 6.87 (4.23-10.78) | 17.20 (13.99-23.77) | Kruskal-Wallis | <0.001 |
| **Energy-adjusted NEAP (mEq∕day)** | 42.70 (38.50-47.44) | 50.55 (47.90-55.25) | 57.98 (54.53-63.01) | 75.82 (66.14-82.44) | Kruskal-Wallis | <0.001 |

| **Table S2: Median (IQR) values of age, physical activity, CAD severity (Gensini score), metabolic, and clinical parameters, across quartiles of Net Endogenous Acid Production (NEAP) in a cross-sectional study.** | | | | | | | |
| --- | --- | --- | --- | --- | --- | --- | --- |
|  |  | **Quartiles of NEAP** | | | | **Test** | **p-value** |
|  |  | **Q1** | **Q2** | **Q3** | **Q4** |  |  |
|  |  | **N=224** | **N=224** | **N=224** | **N=223** |  |  |
| Age (y) |  | 58.00 (51.00-66.00) | 58.00 (50.00-65.50) | 57.50 (51.00-65.00) | 59.00 (52.00-66.00) | Kruskal-Wallis | 0.83 |
| Physical activity (metabolic equivalent minutes/day) |  | 25.00 (10.00-50.00) | 15.00 (5.00-47.50) | 25.00 (10.00-50.00) | 25.00 (10.00-50.00) | Kruskal-Wallis | 0.26 |
| Gensini Score |  | 32.50 (11.00-68.25) | 43.50 (15.50-86.00) | 56.25 (21.00-91.50) | 53.00 (44.00-84.00) | Kruskal-Wallis | <0.001 |
| BMI (kg/m^2^) |  | 27.80 (24.79-32.19) | 27.18 (24.89-29.74) | 25.99 (23.85-29.38) | 26.67 (24.75-29.64) | Kruskal-Wallis | 0.009 |
| Fasting blood sugar (mg/dL) |  | 112.00 (97.50-149.50) | 116.00 (99.00-155.00) | 110.00 (98.50-155.00) | 115.00 (100.00-149.00) | Kruskal-Wallis | 0.59 |
| Cholesterol (mg/dL) |  | 159.00 (131.50-192.00) | 150.00 (128.50-179.50) | 151.00 (129.50-184.00) | 149.00 (130.00-183.00) | Kruskal-Wallis | 0.32 |
| Triglyceride (mg/dL) |  | 140.00 (100.50-195.00) | 142.00 (104.50-211.00) | 142.00 (106.50-197.00) | 142.00 (103.00-191.00) | Kruskal-Wallis | 0.90 |
| eGFR (mL/min/1.73 m²) |  | 70.86 (56.99-87.35) | 71.47 (60.04-83.70) | 70.76 (59.26-86.90) | 73.18 (61.01-86.08) | Kruskal-Wallis | 0.74 |
| Creatinine (mg/dL) |  | 0.94 (0.80-1.13) | 0.95 (0.85-1.12) | 0.93 (0.80-1.10) | 0.95 (0.81-1.09) | Kruskal-Wallis | 0.52 |
| Blood urea nitrogen (BUN) (mg/dL) |  | 15.00 (13.00-18.00) | 16.00 (14.00-19.00) | 15.00 (13.00-18.00) | 15.00 (13.00-18.00) | Kruskal-Wallis | 0.068 |
| Energy-adjusted PRAL (mEq∕day) |  | -9.06 (-15.00--4.68) | -0.08 (-3.09-2.70) | 6.72 (3.91-11.08) | 17.04 (13.62-23.61) | Kruskal-Wallis | <0.001 |
| Energy-adjusted NEAP (mEq∕day) |  | 42.02 (38.47-46.00) | 50.66 (48.42-53.87) | 58.54 (55.55-62.03) | 76.08 (71.39-82.62) | Kruskal-Wallis | <0.001 |

| **Table S3: Median (IQR) values of dietary intakes, across quartiles of energy-adjusted Potential Renal Acid Load (PRAL) in a cross-sectional study.** | | | | | | |
| --- | --- | --- | --- | --- | --- | --- |
|  |  |  |  |  |  |  |
|  | **Quartiles of PRAL** | | | | **Test** | **p-value** |
|  | Q1 | Q2 | Q3 | Q4 |  |  |
|  | **N=224** | **N=224** | **N=224** | **N=223** |  |  |
| Energy (Kcal/day) | 3289.54 (2866.31-3808.09) | 3022.74 (2634.51-3559.06) | 3048.25 (2595.33-3465.88) | 3371.65 (2974.20-3800.74) | Kruskal-Wallis | <0.001 |
| Protein (g/day) | 99.65 (82.74-113.45) | 92.80 (80.72-106.45) | 92.02 (79.82-107.75) | 105.34 (93.72-122.19) | Kruskal-Wallis | <0.001 |
| Carbohydrate (g/day) | 472.38 (383.56-551.82) | 407.29 (344.98-512.58) | 406.55 (331.62-490.76) | 471.30 (392.70-545.94) | Kruskal-Wallis | <0.001 |
| Fat (g/day) | 110.72 (92.13-124.32) | 105.35 (88.90-123.09) | 104.13 (90.38-122.11) | 109.50 (95.50-128.61) | Kruskal-Wallis | 0.040 |
| Saturated fatty acid (g/day) | 47.16 (38.28-53.85) | 45.49 (38.17-53.56) | 44.81 (39.38-53.39) | 48.76 (42.77-56.69) | Kruskal-Wallis | <0.001 |
| Magnesium (mg/day) | 343.47 (302.65-392.43) | 294.79 (256.42-336.11) | 274.09 (243.74-312.16) | 266.20 (229.28-310.73) | Kruskal-Wallis | <0.001 |
| Phosphorus (mg/day) | 1249.42 (1089.72-1448.93) | 1149.81 (997.32-1319.26) | 1104.20 (968.85-1264.62) | 1167.91 (999.53-1310.38) | Kruskal-Wallis | <0.001 |
| Potassium (mg/day) | 3907.92 (3433.85-4404.14) | 3220.34 (2916.94-3589.08) | 2843.50 (2554.13-3191.77) | 2656.84 (2371.17-2959.13) | Kruskal-Wallis | <0.001 |
| calcium (mg/day) | 1073.89 (909.14-1248.02) | 989.15 (819.06-1165.36) | 958.01 (822.32-1165.74) | 1033.49 (898.28-1242.90) | Kruskal-Wallis | <0.001 |
| Meat (g/day) | 92.92 (74.92-115.63) | 100.30 (83.82-119.77) | 101.55 (82.47-122.60) | 118.18 (95.66-139.58) | Kruskal-Wallis | <0.001 |
| Egg (g/day) | 11.44 (5.59-18.64) | 12.29 (5.05-19.44) | 12.00 (5.64-18.50) | 13.35 (7.63-20.98) | Kruskal-Wallis | 0.10 |
| Vegetables (g/day) | 341.61 (281.73-421.53) | 287.42 (240.53-348.99) | 257.58 (205.51-306.16) | 225.99 (178.70-281.99) | Kruskal-Wallis | <0.001 |
| Fruits (g/day) | 484.95 (374.13-635.81) | 359.93 (264.57-445.55) | 264.60 (211.26-354.66) | 222.06 (160.75-292.89) | Kruskal-Wallis | <0.001 |
| Grains (g/day) | 645.36 (508.43-813.01) | 646.98 (509.40-789.91) | 680.60 (553.08-833.26) | 812.96 (656.21-966.31) | Kruskal-Wallis | <0.001 |
| Dairy (g/day) | 418.78 (295.90-564.32) | 401.96 (284.55-508.70) | 391.87 (282.74-522.29) | 424.83 (311.50-568.87) | Kruskal-Wallis | 0.19 |

| **Table S4: Median (IQR) values of dietary intakes, across quartiles of Net Endogenous Acid Production (NEAP) in a cross-sectional study.** | | | | | | |
| --- | --- | --- | --- | --- | --- | --- |
|  |  | **Quartiles of NEAP** | | | **Test** | **p-value** |
|  | **Q1** | **Q2** | **Q3** | **Q4** |  |  |
|  | **N=224** | **N=224** | **N=224** | **N=223** |  |  |
| Energy (Kcal/day) | 3190.16 (2794.43-3867.14) | 3049.71 (2658.35-3520.62) | 3033.15 (2627.90-3392.36) | 3449.63 (3011.08-3844.23) | Kruskal-Wallis | <0.001 |
| Protein (g/day) | 96.89 (80.88-114.18) | 94.44 (81.19-109.83) | 92.74 (83.21-105.79) | 106.16 (93.92-121.24) | Kruskal-Wallis | <0.001 |
| Carbohydrate (g/day) | 457.46 (369.88-560.00) | 414.34 (345.19-505.28) | 396.07 (336.66-464.00) | 486.28 (405.37-555.92) | Kruskal-Wallis | <0.001 |
| Fat (g/day) | 112.48 (91.82-128.29) | 105.78 (92.65-123.96) | 104.54 (90.52-120.55) | 107.34 (93.40-125.95) | Kruskal-Wallis | 0.13 |
| Saturated fatty acid (g/day) | 46.75 (37.30-54.64) | 46.01 (38.80-54.94) | 44.62 (39.10-53.32) | 48.68 (42.67-55.53) | Kruskal-Wallis | 0.005 |
| Magnesium (mg/day) | 348.94 (309.60-409.24) | 298.13 (267.14-340.26) | 283.88 (247.35-316.10) | 256.23 (221.85-285.63) | Kruskal-Wallis | <0.001 |
| Phosphorus (mg/day) | 1276.59 (1101.35-1497.35) | 1169.78 (1004.94-1343.62) | 1130.91 (995.49-1276.57) | 1092.56 (940.17-1234.53) | Kruskal-Wallis | <0.001 |
| Potassium (mg/day) | 3940.81 (3403.15-4487.88) | 3271.59 (2935.84-3674.39) | 2888.63 (2623.38-3246.03) | 2610.77 (2331.96-2909.74) | Kruskal-Wallis | <0.001 |
| calcium (mg/day) | 1074.87 (910.59-1255.53) | 1034.29 (844.48-1199.97) | 953.44 (820.80-1167.65) | 1007.29 (876.09-1201.72) | Kruskal-Wallis | <0.001 |
| Meat (g/day) | 90.39 (71.34-113.43) | 99.05 (83.44-121.89) | 107.82 (86.05-129.98) | 111.95 (94.68-132.04) | Kruskal-Wallis | <0.001 |
| Egg (g/day) | 11.44 (4.00-18.62) | 11.57 (5.68-20.37) | 12.58 (5.34-19.07) | 12.34 (7.63-19.07) | Kruskal-Wallis | 0.20 |
| Vegetables (g/day) | 337.04 (267.30-415.06) | 294.75 (237.89-351.81) | 264.86 (210.32-311.15) | 222.97 (177.07-279.88) | Kruskal-Wallis | <0.001 |
| Fruits (g/day) | 450.91 (352.72-613.39) | 362.18 (273.42-446.13) | 266.16 (207.81-365.84) | 229.78 (167.71-300.08) | Kruskal-Wallis | <0.001 |
| Grains (g/day) | 642.78 (502.20-815.90) | 652.07 (516.67-803.35) | 654.59 (552.52-826.94) | 812.96 (669.71-977.49) | Kruskal-Wallis | <0.001 |
| Dairy (g/day) | 412.42 (293.99-548.51) | 427.42 (305.86-550.08) | 398.34 (284.60-519.61) | 399.58 (294.16-545.46) | Kruskal-Wallis | 0.39 |

| **Table S5. Odds Ratios (ORs) and 95% Confidence Intervals (95% CIs) of Severe Coronary Artery Disease (defined as Gensini Score ≥40) According to Quartiles of Dietary Acid Load Indices.** * | | | | | |
| --- | --- | --- | --- | --- | --- |
|  |  | **Quartiles** | | | **P for trend** |
|  | **1st (Ref)** | **2nd** | **3rd** | **4th** |  |
| **Quartiles of Energy-Adjusted Potential Renal Acid Load (PRAL) (mEq/day)** | | | | | |
| **Median (mEq/day)** | -8.67 | 0.95 | 8.51 | 16.91 |  |
| **Non-cases / Cases** | 127 / 97 | 99 / 125 | 87 / 137 | 57 / 166 |  |
| **Crude model** | 1.00 (Ref) | 1.65 (1.14–2.40) | 2.06 (1.41–3.01) | 3.81 (2.55–5.69) | **<0.001** |
| **Model a** (Age + energy) | 1.00 (Ref) | 2.18 (1.46–3.25) | 2.84 (1.89–4.26) | 4.04 (2.65–6.14) | **<0.001** |
| **Model b** (Fully adjusted) | 1.00 (Ref) | 2.14 (1.35–3.39) | 3.62 (2.28–5.77) | 4.39 (2.70–7.15) | **<0.001** |

**Quartiles of Energy-Adjusted Net Endogenous Acid Production (NEAP) (mEq/day)**

| **Median (mEq/day)** | 42.79 | 52.25 | 60.11 | 74.18 |  |
| --- | --- | --- | --- | --- | --- |
| **Non-cases / Cases** | 137 / 87 | 110 / 114 | 82 / 142 | 41 / 182 |  |
| **Crude model** | 1.00 (Ref) | 1.63 (1.12–2.38) | 2.73 (1.86–4.00) | 6.99 (4.54–10.77) | **<0.001** |
| **Model a** (Age + energy) | 1.00 (Ref) | 2.13 (1.42–3.18) | 3.98 (2.63–6.04) | 7.67 (4.86–12.09) | **<0.001** |
| **Model b** (Fully adjusted) | 1.00 (Ref) | 2.02 (1.26–3.24) | 5.11 (3.15–8.28) | 8.98 (5.28–15.29) | **<0.001** |
| - Model ^a^: Adjusted for age and total energy intake. - Model ^b^: Further adjusted for BMI (kg/ m²) eGFR (mL/min/1.73 m²), gender, smoking, opium use, education, LVEF category, hypertension, prediabetes/T2DM, dyslipidemia, anticoagulant use, anti-inflammatory drugs use, anti-hyperlipidemic use, antidiabetics use, and physical activity.   Ref: Reference category. | | | | | |

*Almost consistency with the main analysis in which severe CAD is defined as Gensini Score ≥60.

| **Table S6. Odds Ratios (ORs) and 95% Confidence Intervals (95% CIs) of Severe Coronary Artery Disease (defined as Gensini Score ≥50) According to Quartiles of Dietary Acid Load Indices*** | | | | | |
| --- | --- | --- | --- | --- | --- |
|  |  | **Quartiles** | | | **P for trend** |
|  | **1st (Ref)** | **2nd** | **3rd** | **4th** |  |
| **Quartiles of Energy-Adjusted Potential Renal Acid Load (PRAL) (mEq/day)** | | | | | |
| **Median (mEq/day)** | -8.67 | 0.95 | 8.51 | 16.91 |  |
| **Non-cases / Cases** | 139 / 85 | 114 / 110 | 113 / 111 | 90 / 133 |  |
| **Crude model** | 1.00 (Ref) | 1.58 (1.08–2.30) | 1.61 (1.10–2.34) | 2.42 (1.65–3.53) | **<0.001** |
| **Model a** (Age + energy) | 1.00 (Ref) | 1.77 (1.21–2.61) | 1.81 (1.23–2.67) | 2.40 (1.64–3.53) | **<0.001** |
| **Model b** (Fully adjusted) | 1.00 (Ref) | 1.69 (1.08–2.66) | 2.21 (1.41–3.47) | 2.25 (1.43–3.54) | **<0.001** |

**Quartiles of Energy-Adjusted Net Endogenous Acid Production (NEAP) (mEq/day)**

| **Median (mEq/day)** | 42.79 | 52.25 | 60.11 | 74.18 |  |
| --- | --- | --- | --- | --- | --- |
| **Non-cases / Cases** | 146 / 78 | 119 / 105 | 102 / 122 | 89 / 134 |  |
| **Crude model** | 1.00 (Ref) | 1.65 (1.13–2.42) | 2.24 (1.53–3.28) | 2.82 (1.92–4.14) | **<0.001** |
| **Model a** (Age + energy) | 1.00 (Ref) | 1.86 (1.26–2.75) | 2.63 (1.77–3.90) | 2.79 (1.89–4.12) | **<0.001** |
| **Model b** (Fully adjusted) | 1.00 (Ref) | 1.78 (1.13–2.83) | 3.23 (2.03–5.11) | 2.44 (1.54–3.88) | **<0.001** |
| - Model ^a^: Adjusted for age and total energy intake. - Model ^b^: Further adjusted for BMI (kg/ m²) eGFR (mL/min/1.73 m²), gender, smoking, opium use, education, LVEF category, hypertension, prediabetes/T2DM, dyslipidemia, anticoagulant use, anti-inflammatory drugs use, anti-hyperlipidemic use, antidiabetics use, and physical activity.   Ref: Reference category. | | | | | |

*Almost consistency with the main analysis in which severe CAD is defined as Gensini Score ≥60.
